# An Unusual Presentation of Large B-cell Lymphoma

**DOI:** 10.7759/cureus.6180

**Published:** 2019-11-18

**Authors:** Joel Robinette, Chris White

**Affiliations:** 1 Otolaryngology / Surgery, West Virginia School of Osteopathic Medicine, Lewisburg, USA; 2 Otolaryngology, Greenbrier Valley Medical Center, Fairlea, USA

**Keywords:** dacryocystitis, lymphoma, lacrimal sac tumors, diffuse large b-cell lymphoma

## Abstract

Primary Non-Hodgkin's lymphoma of the lacrimal sac is extremely rare. Symptoms are usually atypical and nonspecific, which often leads to the original misdiagnosis of dacryocystitis. The most common presenting features are epiphora, swelling, and acute dacryocystitis. We present a case of a 67-year-old female with primary diffuse large B-cell lymphoma (DLBCL) of the lacrimal sac, which was originally diagnosed as dacryocystitis. This case report adds to the urgency that prompt and precise diagnosis and treatment is key.

## Introduction

Malignancy of the lacrimal sac is rare; 6% of which are lymphomas [1]. In the immunocompetent, primary lymphoma occurs at a rate of 0.3% per 100,000 persons [2]. Of all primary lacrimal sac tumors, primary diffuse large B cell lymphoma (DLBCL) occurs at 43%, MALToma at 24%, unclassified B-cell lymphoma at 21%, lymphoid hyperplasia at 5%, and each small lymphocytic lymphoma and natural killer (NK)/T-cell lymphoma at 3% [3]. One of the most common presenting features of DLBCL is dacryocystitis, which is a blockage of the nasolacrimal duct leading to an infection of the lacrimal sac [3]. The symptoms of DLBCL are usually atypical and nonspecific, which often leads to the original diagnosis with dacryocystitis [4-5]. The most presenting features are epiphora (96%), swelling in the lacrimal sac region (75%), and acute dacryocystitis (31%) [3]. Patients with primary lacrimal sac tumors are often older and female [6].

We present a case of a 67-year-old woman with DLBCL of the lacrimal sac who was originally diagnosed with dacryocystitis. She presented with an overflow of tears onto the face (epiphora), swelling, and intermittent pressure on the left eye. Herein, we highlight the importance of early diagnosis and treatment for atypical presentations of lymphoma.

## Case presentation

**Figure 1:**
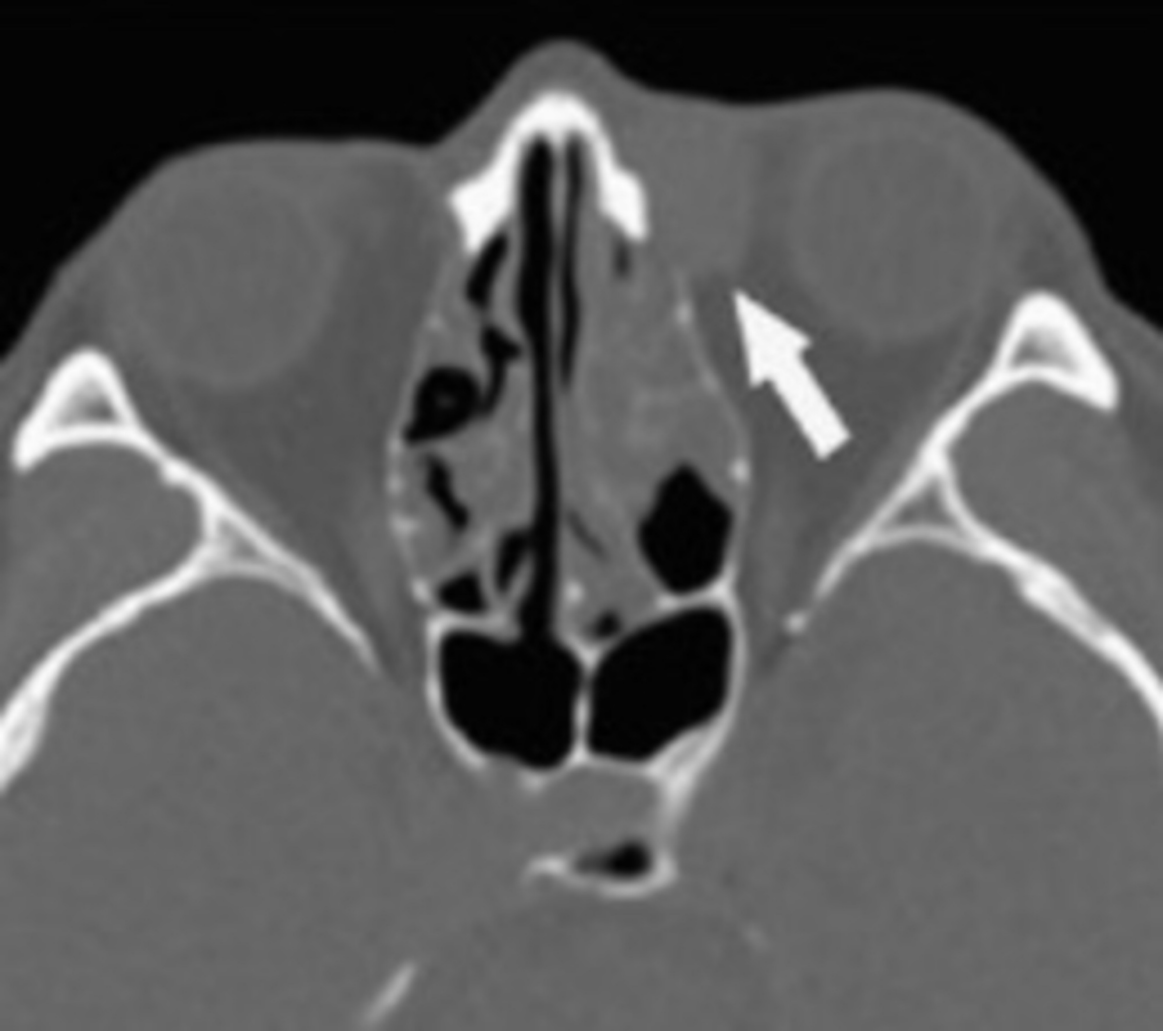
Computed tomography demonstrating homogeneous soft tissue density in the medial surface of the globe indicated by the tip of the arrow.

A 67-year-old female presented with a raised and pruritic ovoid swelling located left of the medial canthus and noted excessive tearing from her left tear duct. She was seen by her ophthalmologist who probed and irrigated her canaliculi with a steroid and antibiotic in late November 2018. Her epiphora improved, however, she noted a pea-size mass and thickening which remained. Non-contrast computed tomography (CT) of the paranasal sinuses on December 19, 2018, showed an 8x10x14 mm ovoid, well-circumscribed collection of soft tissue in the left medial canthus involving the lacrimal drainage apparatus as seen in Figure 1 [7]. She reported intermittent pressure in the left eye on December 27, 2018.

**Figure 2:**
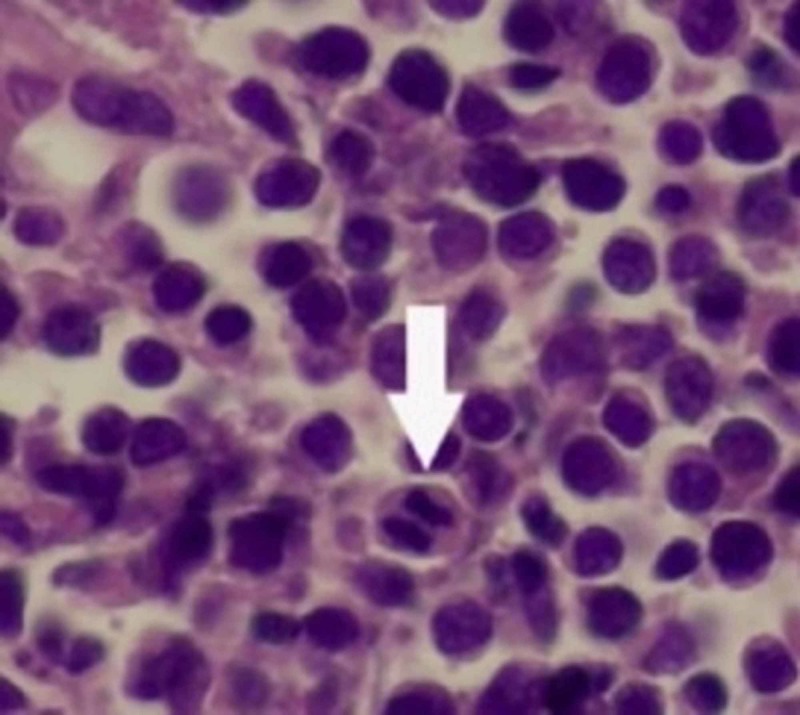
Histopathological studies with hematoxylin and eosin stain demonstrate neoplastic cells with high mitotic activity and variable size.

She then underwent endoscopic dacryocystorhinostomy on January 14, 2019, and was found to have what was thought to be a dacryocystocele, which was subsequently biopsied. Pathology of the dacryocyst was concerning for large B-cell malignant lymphoma as seen in Figure 2 [7]. The specimen was cluster of differentiation 45 (CD45) positive, S100 negative, A/E ⅓ essentially negative, B-cell leukemia/lymphoma 1 (BCL1) negative, BCL2 scattered nondescript positivity, BCL6 scattered positivity, CD3 scattered positivity in T cells, CD5 positive in T cells, CD20 positive, CD23 essentially negative, CD43 positive in T cells, and CD79a positive in large atypical cellular infiltrate. nodular lymphocyte-predominant Hodgkin lymphoma (MUM1) was negative, and Ki67 was ~80%. The slides were then reviewed at the University of Virginia Health System University Hospitals labs, which again read as markedly atypical large cell infiltrate favor large cell malignant lymphoma. 

The patient completed staging with positron emission tomography-computed tomography (PET/CT), had subsequent bone marrow biopsies with no bone marrow involvement, and labs followed by prognosis. She is currently receiving chemotherapy regimens and has remained stable since her last office visit with her otolaryngologist.

## Discussion

Primary Non-Hodgkin's lymphoma of the lacrimal sac is extremely rare, and patients are often older and female [6]. The symptoms of DLBCL are usually atypical and nonspecific, which often leads to the original misdiagnosis of dacryocystitis [4-5]. The most presenting features are epiphora (96%), swelling in the lacrimal sac region (75%), and acute dacryocystitis (31%) [3]. Our patient presented with features of epiphora, which was improved after steroid and antibiotic treatment by her ophthalmologist. She also presented with swelling and intermittent pressure on the left eye.

In a literature review of 17 case-control studies, a total of 3865 histopathologically examined lacrimal sac wall biopsy specimens from 3662 patients taken during dacryocystorhinostomy were analyzed, and it was found that 45% of primary lacrimal sac malignant neoplasms were not suspected [8]. This evidence highlights the importance of early diagnosis and the significance of routine biopsy during dacryocystorhinostomy for suspected dacryocystitis. Our patient was originally diagnosed with dacryocystitis and received prompt dacryocystorhinostomy, which was subsequently biopsied without any particular concern for malignancy. Prompt and precise diagnosis occurred in <15% in some cases while others were unintentionally diagnosed during other procedures [9-10]. Our patient was originally suspected to have dacryocystitis and was diagnosed with large B-cell lymphoma two months later. This demonstrated the potential urgency for early diagnosis and treatment for possible malignancy of the lacrimal gland.

Most cases involve possible surgical resection, chemotherapy, and/or radiation. This management involved a high success rate with local disease control; however, with systemic disease involvement, 15% died after 18 months [3]. After surgical resection, our patient began receiving chemotherapy treatment and has been stable as of her last office visit.

## Conclusions

Malignant lymphoma of the lacrimal gland are rare and can easily imitate dacryocystitis. We present a case of a 67-year-old woman with primary diffuse large B-cell lymphoma (DLBCL) of the lacrimal sac who was originally diagnosed with dacryocystitis. She presented with features of epiphora, swelling, and intermittent pressure on the left eye. Due to the infiltrating nature of these tumors and differential variants, early diagnosis and treatment is essential to optimize outcomes. This case report adds to the urgency that prompt and precise diagnosis and treatment are key.
